# SNAP23 decreases insulin secretion by competitively inhibiting the interaction between SNAP25 and STX1A

**DOI:** 10.1042/BSR20222594

**Published:** 2023-04-28

**Authors:** Jun Chen, Ziyan Wang, Tuanlao Wang, Jidong Cheng, Ruijuan Zhuang, Wei Wang

**Affiliations:** 1 Department of Endocrinology, Xiang’an Hospital of Xiamen University, School of Medicine, Xiamen University, Xiamen 361104, China; 2 School of Pharmaceutical Sciences, State Key Laboratory of Cellular Stress Biology, Fujian Provincial Key Laboratory of Innovative Drug Target Research, Xiamen University, Xiamen 361104, China

**Keywords:** β cell, insulin, SNAP23, snap25, SNARE

## Abstract

SNAP25 is a core protein of the SNARE complex, which mediates stimulus-dependent secretion of insulin from the pancreatic β cells. SNAP23 is a SNAP25 homolog, however, the functional role of SNAP23 in the exocytic secretion of insulin is not known. Therefore, in the present study, we investigated the functional role of SNAP23 in the insulin secretory pathway. Our results demonstrated that over-expression of SNAP23 inhibited the secretion of insulin from the INS-1 cells. Conversely, SNAP23 depletion increased insulin secretion. Mechanistically, overexpression of SNAP23 decreased SNARE complex formation by blocking the binding of SNAP25 to STX1A. The full-length SNAP23 protein with the N-terminal and C-terminal SNARE binding domains was required for competition. Moreover, SNAP23 serine 95 phosphorylation plays a crucial function in insulin secretion by enhancing the interaction between SNAP23 and STX1A. The present study presents a new pathway regulating insulin secretion. Therefore, SNAP23 may be a potential therapeutic target for diabetes mellitus.

## Introduction

SNARE proteins regulate the release of neurotransmitters, insulin, and other secretory proteins by mediating the fusion of secretory vesicles with the plasma membranes of the target cells [1–11]. The membrane fusion is facilitated by the formation of a quaternary complex between Sec1/Munc18-like (SM), two t-SNARE proteins (syntaxin and SNAP), and the v-SNARE protein, VAMP [12–14].

The three different types of secretory granules (SGs) involved in glucose-stimulated insulin secretion (GSIS) are as follows: (1) pre-docked SGs: the SGs are already anchored on the plasma membrane (PM) under basal conditions and are secreted upon nutritional stimulus; (2) newcomer SGs: the SGs are not anchored on the PM or are only temporarily anchored on the PM under basal conditions; and (3) SG-SG fusion [15–22]. In the pre-docked SGs, SNAP25 is required for the formation of SNARE complex through interaction with Munc18a, STX1A, and VAMP2. SNARE complex mediates fusion of pre-docked insulin SGs within a rapidly releasable pool; the formation of new SGs is mediated by the interactions between SNAP25, Munc18b, STX-3, and VAMP8 proteins; SG-SG fusion has not been investigated extensively and accounts for approximately 3% of the total insulin secretion [23–25].

Approximately 60 SNARE proteins have been reported in mammalian cells [26]. SNARE proteins demonstrate unique expression patterns in the organs, intracellular localization, and SNARE complex partners [27–30]. SNAP23 is a SNAP25 homolog that is expressed in a variety of exocrine and endocrine cells and participates in the exocytosis process [31–34]. SNAP23 is implicated in the exosomal secretory process in hepatocellular carcinoma, secretion of zymogen granules from the pancreatic exocrine cells, and secretion of pre-stored inflammatory mediators from the mast cells [35–37]. In the present study, we investigated the functional role of SNAP23 in the secretion of insulin from pancreatic β cells.

## Results

### SNAP23 overexpression inhibits secretion of insulin from the pancreatic β cells

**Figure 1:**
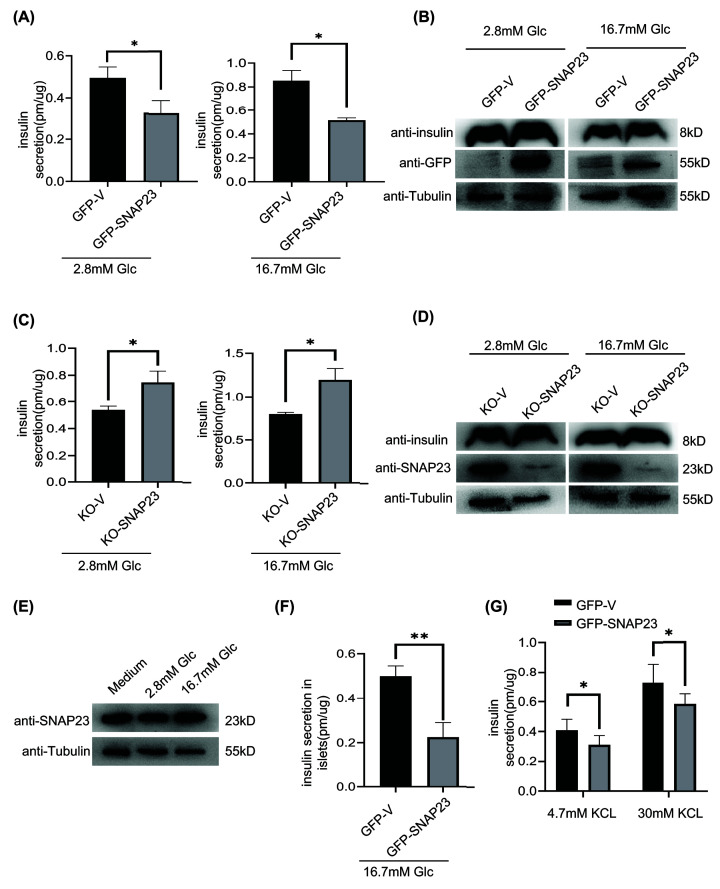
SNAP23 overexpression inhibits insulin secretion in the rat pancreatic β cells (**A**) INS-1 cells were infected with lentiviruses carrying GFP-Vector or GFP-SNAP23 constructs for 48 h. The transfected cells were treated with 2.8 mM glucose in KRBH buffer for 1 h. Then, the cells were stimulated with 16.7 mM glucose in KRBH buffer for 30 min. The insulin content in the supernatants were assessed by ELISA. (**B**) Western blotting analysis of total cellular protein extracts from (**A**) for SNAP23, insulin, and tubulin. (**C**) ELISA results show insulin concentrations in the supernatant of INS-1 cells infected with lentiviruses carrying KO-Vector or KO-SNAP23 constructs, incubated with 2.8 mM glucose in KRBH buffer for 1 h, and subsequently stimulated with 2.8 or 16.7 mM glucose in KRBH buffer for 30 min. (**D**) Western blotting analysis of total cellular protein extracts from (C) for SNAP23, insulin, and tubulin. (**E**) INS-1 cells treated with medium, 2.8 or 16.7 mM glucose in KRBH buffer for 6 h. (**F**) ELISA results show insulin concentrations in the supernatant of mouse islets infected with lentiviruses carrying GFP-Vector or GFP-SNAP23 constructs for 48 h, incubated with 2.8 mM glucose in KRBH buffer for 1 h, and subsequently stimulated with 16.7 mM glucose in KRBH buffer for 30 min. (**G**) ELISA results show insulin concentrations in the supernatant of INS-1 cells infected with lentiviruses carrying GFP-Vector or GFP-SNAP23 constructs for 48 h, incubated with 2.8 mM glucose in KRBH buffer for 1 h, and subsequently stimulated with 4.7 or 30 mM KCl in KRBH buffer for 30 min; *n*=3, *P<0.05, **P<0.01.

**Figure 2:**
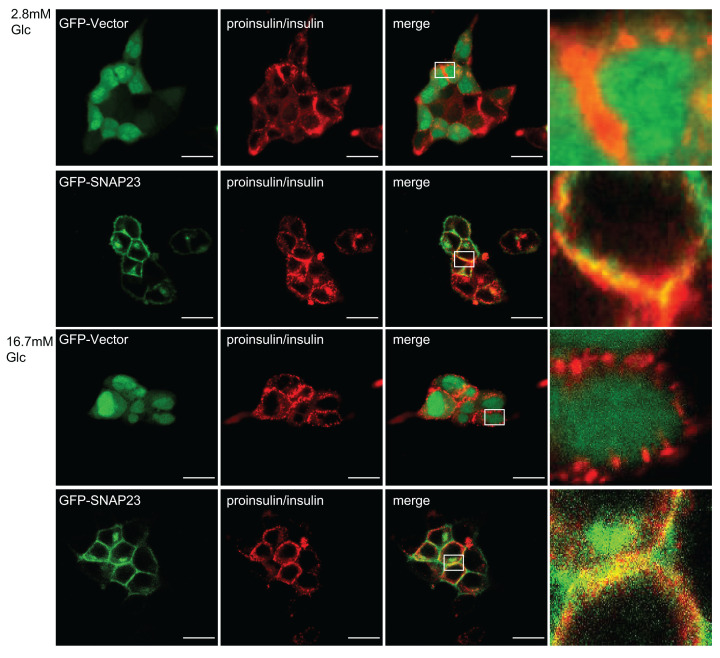
SNAP23 co-localizatize with insulin in the INS-1 cells Representative confocal fluorescence microscopy images show immunofluorescence labeling of insulin with a fluorescence-conjugated antibody (red) in the GFP-Vector- and GFP-SNAP23-expressing INS-1 cells that were incubated with 2.8 mM (top two lines)or 16.7 mM glucose in KRBH buffer for 30 min.

We first investigated the role of SNAP23 in the secretion of insulin from the pancreatic β cells. Therefore, SNAP23 was overexpressed in the INS-1 rat pancreatic β-cell line using lentiviral vectors. Insulin secretion was measured in the control and SNAP23-overexpressing INS-1 cells after stimulation with 2.8 and 16.7 mM glucose for 30 min. Insulin secretion was significantly reduced in the SNAP23-overexpressing INS-1 cells compared with the corresponding controls (Figure 1A,B). In contrast, SNAP23 knockdown increased insulin secretion from INS-1 cells stimulation with 2.8 and 16.7 mM glucose (Figure 1C,D). SNAP23 protein level unchanged in 2.8 and 16.7 mM glucose stimulation condition (Figure 1E). Freshly isolated mouse islets were effectively infected with lentivirus carrying GFP-SNAP23 and then stimulated by 16.7 mM glucose. Again, the SANP23 overexpression significantly inhibited insulin secretion in islets (Figure 1F). SNAP23 overexpression also reduced insulin secretion from INS-1 cells treated with 4.7 and 30 mM KCl (Figure 1G). Furthermore, immunofluorescence data showed co-localization of SNAP23 with insulin in the INS-1 cells stimulated with 2.8 and 16.7 mM glucose (Figure 2). These findings suggested that insulin secretion from β cells was inhibited by SNAP23 overexpression.

### SNAP23 inhibits insulin secretion by blocking the binding of SNAP25 to STX1A

**Figure 3:**
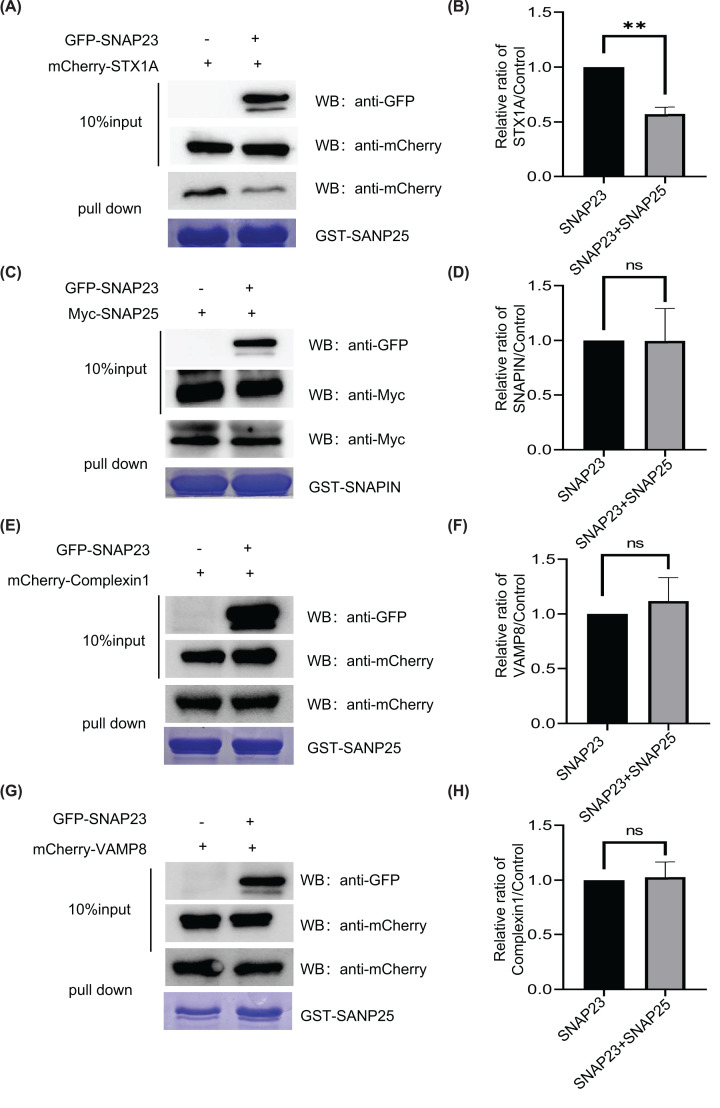
SNAP23 inhibits secretion of insulin by blocking the binding of SNAP25 to STX1A (**A,C,E,G**) Western blotting analysis shows the results of the pull-down assay. 293T cells were co-transfected with SNAP23 and STX-1A (A), SNAP25 (C), Complexin1 (E) and VAMP8 (G) for 48 h. Then, total cell extracts were prepared and STX1A, SNAP25, Complexin 1, and VAMP8 using GST-SNAP25 or GST-SNAPIN (C). (**B,D,F,H**) The normalized histogram plots show the relative expression levels of STX-1A, SNAPIN, Complexin 1, and VAMP8 proteins compared with the corresponding controls using the ImageJ software; *n*=3, **P<0.01. ns: no significance.

Based on the amino acid sequence, SNAP23 is 59% identical to SNAP25 [38]. Furthermore, SNAP23 interacts with several Qa-SNAREs, including syntaxin-1a [39]. Moreover, SNAP-23 interacts with R-SNAREs such as VAMP8 [40]. This suggested that SNAP23 may competed with SNAP25 for binding to the SNARE complex proteins. To test this hypothesis, we simultaneously transfected 293T cells with GFP-tagged SNAP23 and mCherry-tagged SNARE proteins (STX1A, VAMP8, Complexin I, and Snapin), and performed pull-down experiments with GST-tagged SNAP25. Western blot results demonstrated that SNAP23 overexpression reduced the interaction between SNAP25 and STX1A (Figure 3A,B). However, SNAP23 did not influence the interaction between SNAP25 and VAMP8, Complexin I, or Snapin (Figure 3C–H). These findings suggested that SNAP23 inhibited insulin secretion by competing with SNAP25 for binding to STX1A.

### Full-length wild-type SNAP23 is required for inhibiting insulin secretion

**Figure 4:**
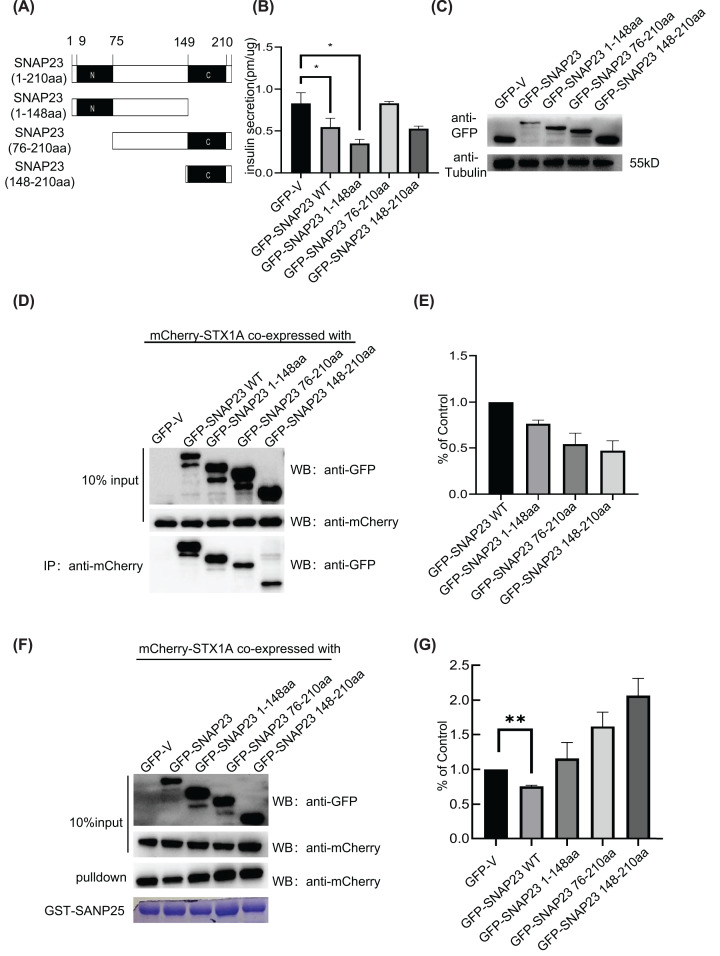
Full-length SNAP23 is required for inhibiting insulin secretion from the INS-1 cells (**A**) The domain map of SNAP23 indicates the N-terminal domain (amino acids 9-75) and C-terminal domain (amino acids: 149–207). The truncated SNAP23 mutants were constructed by deleting specific domains of SNAP23 as indicated. (**B**) ELISA results show the insulin concentrations in the supernatants of INS-1 cells infected with lentiviruses carrying vectors with WT or truncated SNAP23 constructs for 48 h followed by incubation with 2.8 mM glucose in KRBH buffer for 1h and subsequent stimulation with 16.7 mM glucose in KRBH buffer for 30 min. (**C**) Western blotting assay results show the levels of GFP-tagged SNAP23 proteins (WT or truncated) and tubulin in total protein cell extracts from (B). (**D**) 293T cells were co-transfected with GFP-tagged WT or truncated SNAP23 constructs and mCherry-STX 1. The lysates were directly immunoblotted or immunoprecipitated with the antibody against mCherry followed by immunoblotting with the anti-GFP antibody. (**E**) The normalized histograms show the relative levels of co-precipitated truncated SNAP23 proteins against the WT SNAP23 protein as control. (**F**) Pull-down assay results show the levels of GFP-tagged WT or truncated SNAP23 proteins. 293T cells were co-transfected with GFP-tagged WT or truncated SNAP23 constructs and mCherry-STX 1A. GST-SNAP25 was used to pull down STX1A from the total protein extracts. (**G**) The normalized histograms show the levels of STX-1A protein pulled down from 293T cells co-transfected with the GFP-tagged vector control or GFP-tagged WT or truncated SNAP23 constructs using the ImageJ software; *n*=3, *P<0.05, **P<0.01.

Next, we transfected the INS-1 cells with GFP-tagged deletion constructs of SNAP23 to determine the specific domains of SNAP23 that regulate insulin secretion. INS-1 cells were transfected with full-length WT SNAP23 (amino acids 1–210), SNAP23 with only N-terminal SNARE motif and linker region (amino acids 1–148), SNAP23 with only linker region and C-terminal SNARE motif (amino acids 76–210), and SNAP23 with only C-terminal SNARE motif (amino acids 148–210) (Figure 4A). ELISA analysis showed that insulin secretion was not reduced in the INS-1 cells transfected with the SNAP23 (76–210) mutant, but significantly reduced in the INS-1 cells transfected with the SNAP23 (1–148) mutant (Figure 4B). The overexpression efficiency of various SNAP23 mutants is shown in Figure 4C. Both SNAP23 SNARE motifs showed the ability to interact with STX1A, but the interaction of STX1A was stronger with the N terminal SNARE motif compared with the C-terminal SNARE motif (Figure 4D,E). Furthermore, only the full-length SNAP23 competed with SNAP25 for binding STX1A (Figure 4F,G).These results demonstrated that the full-length SNAP23 was required for inhibiting the interaction of SNAP25 with STX1A. Deletion of any one domain in SNAP23 abrogated its efficacy in competing with SNAP25.

### Serine 95 phosphorylation is required for inhibition of insulin secretion by SNAP23

**Figure 5:**
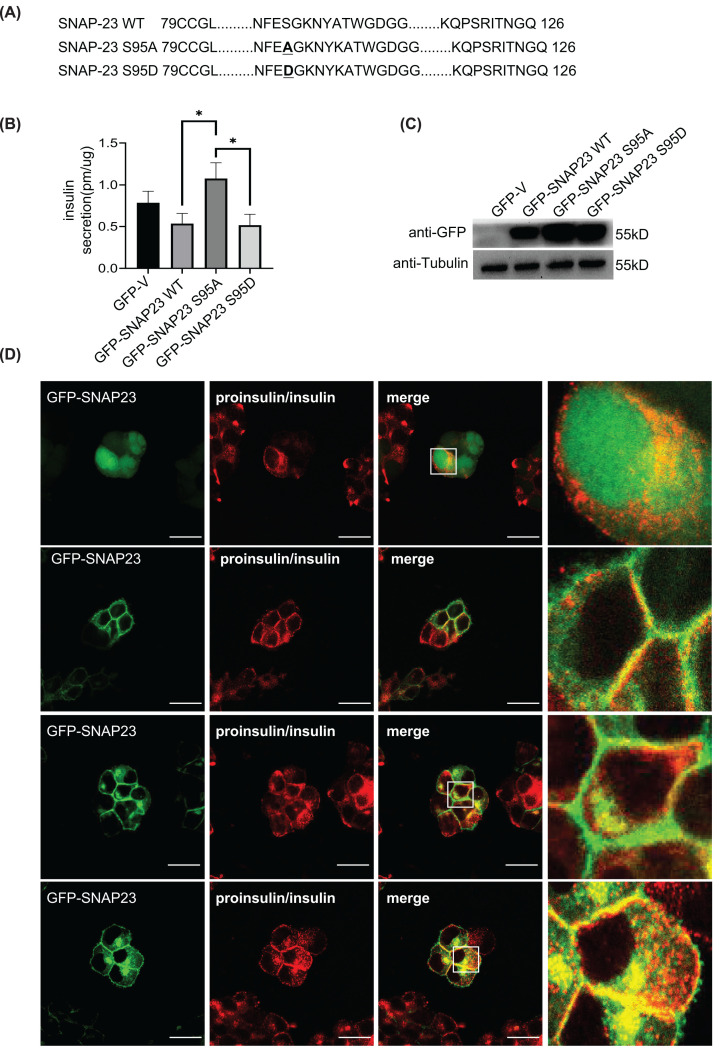
SNAP23 S95 phosphorylation is essential for suppressing insulin secretion (**A**) The sequence alignment of the WT and phosphorylation mutants (S95A/S95D) in the linker region of SNAP23. (**B**) ELISA results show the insulin concentrations in the supernatants of INS-1 cells infected with lentiviruses containing constructs with SNAP23 WT, SNAP23 S95A, or SNAP23 S95D. The cells were incubated with 2.8 mM glucose in the KRBH buffer for 1 h followed by stimulation with 16.7 mM glucose in the KRBH buffer for 30 min. (**C**) Representative western blots show the levels of GFP-SNAP23 (WT or S95A or S95D) and tubulin in the total protein extracts from (**B**). (**D**) Representative images from confocal fluorescence microscopy show the expression and localization of GFP-SNAP23, GFP-SNAP23 S95A, and GFP-SNAP23 S95D in the INS-1 cells stimulated with 16.7 mM glucose in the KRBH buffer for 30 min. The cells were labeled with fluorescence-conjugated antibody against insulin (red); *n*=3, *P<0.05.

**Figure 6:**
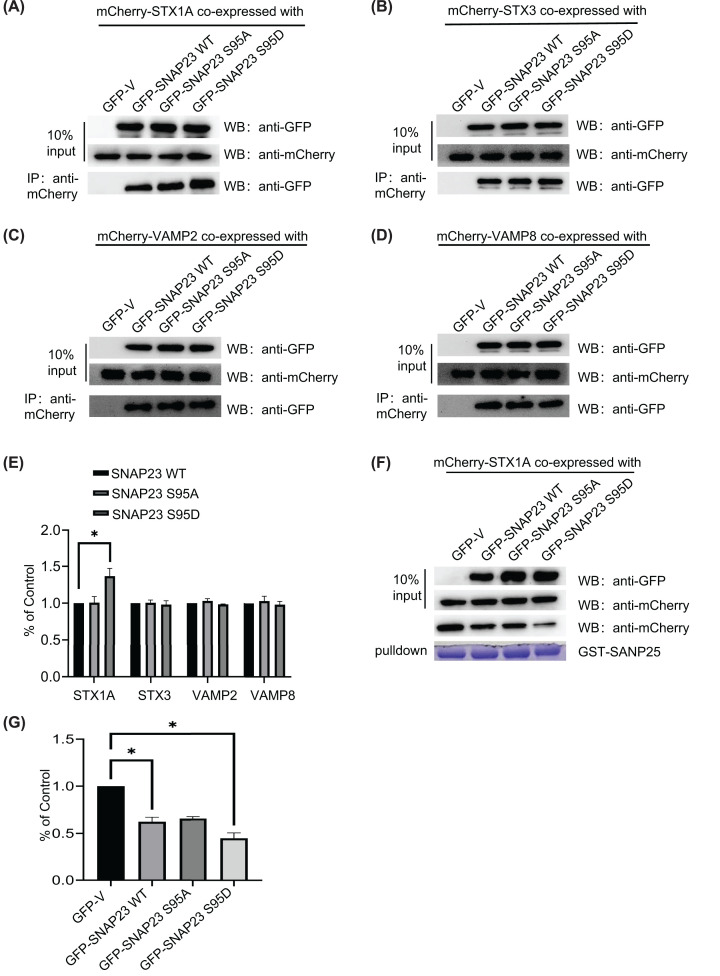
S95 phosphorylation enhanced the interaction between SNAP23 and STX1A (**A–D**) 293T cells were co-transfected with lentiviruses containing constructs with GFP-SNAP23 WT or GFP-SNAP23 S95A, or GFP-SNAP23 S95D and mCherry-STX1A (A), mCherry-STX3 (B), mCherry-VAMP2 (C), or mCherry-VAMP8 (**D**). The cell lysates were directly immunoblotted or immunoprecipitated with the anti-mCherry antibody and then blotted with the anti-GFP antibody. (A) The normalized histograms show the levels of co-precipitated STX1A, STX3, VAMP2, and VAMP8 proteins in cells co-transfected with GFP-SNAP23 S95A, GFP-SNAP23 S95D, or GFP-SNAP23 WT (control) using the ImageJ software. (B) The results of pull-down assay with STX1A. 293T cells were co-transfected with GFP-SNAP23 WT, GFP-SNAP23 S95A, or GFP-SNAP23 S95D, and mCherry-STX1A. The cell extracts were prepared and STX1A was pulled down using GST-SNAP25. (C) The normalized histograms show the levels of SNAP23 protein pulled down in cells co-transfected with GFP-SNAP23 S95A, GFP-SNAP23 S95D, or GFP-SNAP23 WT (control) using the ImageJ software; *n*=3, *P<0.05.

The phosphorylation of serine 95 in SNAP23 by IKK2 is critical for platelet exocytosis [41]. Therefore, we examined if phosphorylation of serine 95 in SNAP23 was required for insulin secretion by mutating the serine (S) amino acid to alanine (A) (SNAP23 S95A) or aspartate (D) (SNAP23 S95D) to abrogate or activate phosphorylation (Figure 5A). Insulin secretion was higher in the INS-1 cells overexpressing SNAP23 S95A and significantly decreased in INS-1 cells overexpressing the SNAP23 S95D mutant (Figure 5B). The overexpression efficiency of SNAP23 phosphorylation mutants is shown in Figure 5C. Furthermore, SNAP23 S95D mutant protein showed strong co-localization with insulin compared with the SNAP23 S95A mutant (Figure 5D). Moreover, the interaction between STX1A and SNAP23 S95D was significantly higher compared with the SNAP23 S95A mutant (Figure 6A–E). However, the interactions of STX3, VAMP2, and VAMP8 with the SNAP23 S95D mutant protein were unaffected (Figure 6B–E). SNAP23 S95D protein also strongly inhibited the interaction between SNAP25 and STX1A (Figure 6F,G). These findings demonstrated that SNAP23 S95 phosphorylation was essential for the release of insulin from the pancreatic β cells.

### T102 and S120 phosphorylation sites are not involved in the inhibition of insulin secretion by SNAP23

**Figure 7:**
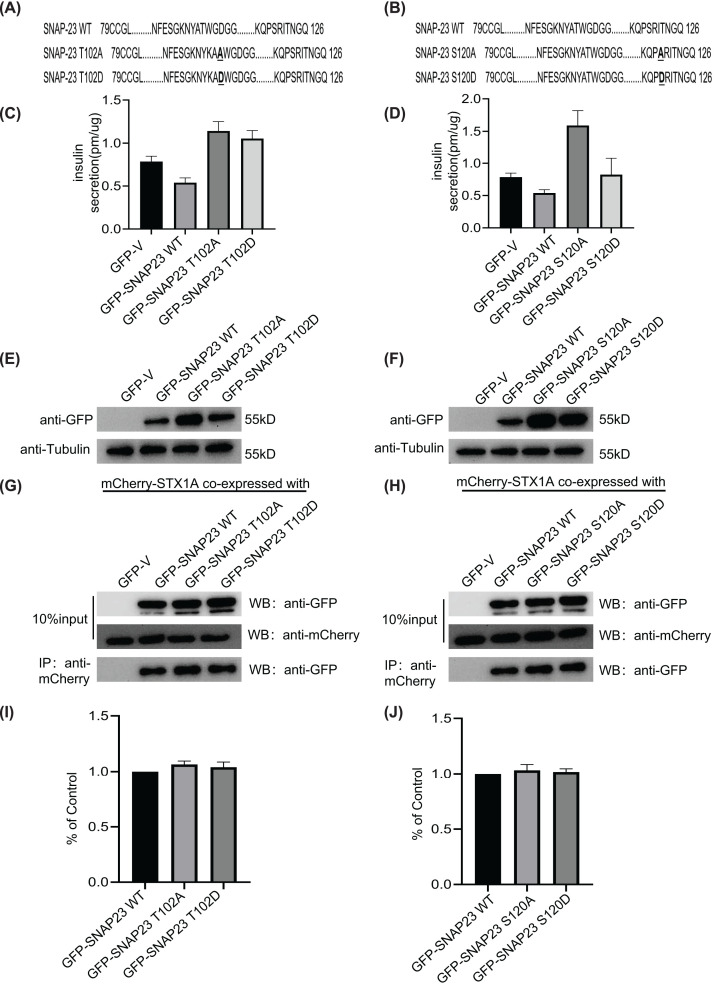
T102 and S120 phosphorylation does not inhibit insulin secretion by SNAP23 (**A,B**) The sequence alignment shows the SNAP-23 WT protein and the SNAP23 phosphorylation mutants (T102A/T102D, S120A/S120D) in the linker region. (**C,D**) ELISA results show the insulin levels in the supernatants from INS-1 cells infected with lentiviruses containing constructs with GFP-SNAP23 WT, GFP-SNAP23 T102A and GFP-SNAP23 T102D (**C**), and GFP-SNAP23 S120A and GFP-SNAP23 S120D (**D**). The cells were incubated with 2.8 mM glucose in the KRBH buffer for 1 h followed by stimulation with 16.7 mM glucose in the KRBH buffer for 30 min. (**E,F**) Western blots show the expression levels of GFP-SNAP23 WT, GFP-SNAP23 T102A, GFP-SNAP23 T102D, GFP-SNAP23 S120A, GFP-SNAP23 S120D, and tubulin in the total protein extracts from (C,D). (**G,H**) 293T cells were co-transfected with GFP-SNAP23 WT, GFP-SNAP23 T102A, GFP-SNAP23 T102D (G) or GFP-SNAP23 S120A and GFP-SNAP23 S120D (H) and mCherry-STX 1A. The lysates were directly immunoblotted or immunoprecipitated with the anti-mCherry antibody and then blotted with the anti-GFP antibody. (**I,J**) The normalized histograms show the levels of the co-precipitated SNAP23 protein in the GFP-SNAP23 WT (control), GFP-SNAP23 T102A, GFP-SNAP23 T102D, GFP-SNAP23 S120A, GFP-and SNAP23 S120D groups using the ImageJ software; *n*=3.

We also analyzed the roles of the T102 and S120 phosphorylation sites in the regulation of insulin secretion by SNAP23 through mutation of threonine (T) to alanine (A) at position 102 (T102A) and S to A at position 120 (S120A) (Figure 7A,B). We did not observe any differences in the insulin secretion rates between INS-1 cells overexpressing SNAP23 T102A and SNAP23 T102D after stimulation with 16.7 mM glucose for 30 min (Figure 7C). However, SNAP23 S120A increased insulin secretion compared with the control, whereas, SNAP23 S120D did not show any difference in the insulin secretion rates compared with the control (Figure 7D). The overexpression efficacy of the SNAP23 phosphorylation mutants are shown in Figure 6E,F. Furthermore, T102D and S120D mutations did not alter the interaction between STX1A and SNAP23 (Figure 7G–J). These findings suggested that T102 and S120 phosphorylation did not significantly alter the insulin secretion rates from the pancreatic β cells.

## Discussion

Diabetes mellitus is a highly prevalent metabolic disorder with serious long-term effects on human health. It is caused by higher glucose levels in blood because of defective insulin production by the pancreatic β cells. Long-term hyperglycemia is associated with multi-organ damage [42]. Since SNAP23 and SNAP25 both form similar SNARE complexes, it is postulated that SNAP23 played a regulatory role in the secretion of insulin from the pancreatic β cells. The present study overexpressed SNAP23 in the INS-1 pancreatic β-cell line and demonstrated that SNAP23 reduced insulin secretion. This suggested a critical regulatory role for SNAP23 in insulin secretion.

Furthermore, this study investigated the mechanism through which SNAP23 regulated insulin secretion. SNAP25 interacted with STX1A, to form a SNARE complex that fuses insulin granules with cell membranes [25].The results showed that SNAP23 competed with SNAP25 for binding to STX1A. Both SNARE-binding motifs of SNAP23 interacted with their partner SNARE protein, STX1A. However, presence of a single SNARE motif was unable to disrupt the formation of a fully functional SNAP25-SNARE complex. This partially explained that observation that the full-length wild-type SNAP23 was required for competitive inhibition of SNAP25 binding to STX1A. However, the present study did not observe any competition between SNAP23 and SNAP25 for binding to VAMP8, another component of the SNARE complexes.

A previous study demonstrated that mutant SNAP25 S187E enhanced insulin secretion in the INS-1 cell line [43]. SNAP-23 homolog is phosphorylated at Ser-95 by PKC. Our study showed that the non-phospho-mimetic mutant, SNAP23 S95A, enhanced insulin secretion. However, the phospho-mimetic mutant, SNAP23 S95D, reduced insulin secretion in the INS-1 cells. The present study further demonstrated that phosphorylation at two other sites, T102 and S120, did not participate in the regulation of insulin secretion in the INS-1 cells. We did not observe any differences in the insulin secretion rates between the T102D and T102A mutants of SNAP23. However, S120A increased insulin secretion but S120D did not affect insulin secretion compared with the wild-type control. This demonstrated that S95 phosphorylation was functionally important for insulin secretion by the pancreatic β cells. Mechanistically, phosphorylation of SNAP23 at Ser95 improved the interaction with STX1A and showed stronger ability to compete with SNAP25. However, T102D and S120D did not alter the interaction between STX1A and SNAP23. The role of SNAP23 Ser-95 requires further investigations in diabetes mellitus and other human diseases because numerous kinases, such as PKC, are activated under pathological and physiological conditions.

The fusion of insulin secretory granules plays a critical role in the maintenance of insulin homeostasis. The present study unravels further mechanistic evidence regarding the formation of SNARE complexes and suggests a potential role for SNAP23 in the pathogenesis of diabetes mellitus. Therefore, SNAP23 can be a potential therapeutic target in diabetes. A previous study by Kunii et al. reported that a small compound called MF286 prevented SNARE complex formation and increased insulin secretion in mice after GSIS by selectively binding to SNAP23 [32]. Further investigations are necessary to unravel the various roles of SNAP23 in the exocrine and endocrine secretory pathways.

## Materials and methods

### Cell culture

The INS-1 rat pancreatic β-cell line and the 293T cell line were purchased from ATCC. INS-1 cells were cultured in RPMI 1640 medium supplemented with 10% FBS, 1 mM sodium pyruvate, and 50 mM β-mercaptoethanol in a humidified incubator at 37°C and 5% CO_2_. 293T cells were cultured in DMEM medium supplemented with 10% FBS in a humidified incubator at 37°C and 5% CO_2_.

### Plasmids for overexpression

The mCherry vector was used to clone STX1A (mCherry-STX1A), Complexin1 (mCherry-Complexin1), and VAMP8 (mCherry-VAMP8) cDNAs. The rat SNAP23 cDNA was cloned into the GFP-C1 vector or pCDH-GFP vector to generate GFP-tagged SNAP23. PCR-based mutagenesis was used to generate SNAP23 domain-defective and phosphorylation-defective mutants.

### CRISPR/Cas9 mediated gene knockout

SNAP23-deficient INS-1 cells were generated using CRISPR/Cas9 system as previously described [44]. sgRNA sequence (5-GGAAAGCACAAGGAGAATCC-3) was used for disrupting the expression of SNAP23 in INS-1 cells.

### Western blotting

Total cellular protein extracts were prepared by lysing cells in the RIPA buffer and protease inhibitor cocktail followed by centrifugation at 12000 rpm for 15 min at 4°C to obtain the supernatants. The total protein concentrations of samples were estimated using the BCA assay kit. Equal amounts of protein extracts were electrophoresed on a 10–15% dodecyl sodium polyacrylamide gel (SDS-PAGE). The separated proteins were transferred on to the polyvinylidene fluoride (PVDF) membranes (Millipore, Billerica, MA). The membranes were blocked with 5% nonfat milk at room temperature for an hour. Then, the membranes were incubated overnight at 4°C with primary antibodies against SNAP23 (Proteintech,Cat 10825-1-AP), Tubulin (Proteintech,cat 66031-1-Ig), insulin(ABclonal, cat A19066), GFP (Proteintech,cat 66002-1-Ig), and mCherry (Proteintech, cat 26765-1-AP). The membranes were washed with phosphate-buffered saline with Tween-20 (PBST) and incubated with the HRP-conjugated secondary antibody in PBST for 1 h at room temperature. The blots were then developed with ECL (Pierce, Thermo Fisher Scientific). The protein bands were quantified using the ImageJ software.

### Confocal immunofluorescence microscopy

Cells were fixed for 30 min at room temperature in 4% paraformaldehyde. Then, the cells were washed three times with cold PBS for 3 min each at room temperature and incubated with blocking solution (5% BSA in PBS) for 1 h at room temperature. The cells were permeabilized with 0.1% Triton X-100 for 10 min and incubated overnight with primary antibodies at 4°C. The cells were rinsed with permeabilization buffer and then incubated with fluorescent-tagged secondary antibodies. The immunolabeled cells were mounted on slides and photographed using a Carl Zeiss LSM5 EXITER laser scanning confocal microscope.

### Immunoprecipitation

293T cells were co-transfected with GFP-SNAP23 and mCherry-STX1A vectors to generate stable cell lines to study the interaction between SNAP23 and STX1A. The cell lysates were immunoprecipitated with anti-mCherry antibodies. 293T cells were homogenized on ice in 500 µl of NP-40 buffer (1% NP-40, 150 mM NaCl, 50 mM Tris Base, 1% Triton X-100) and a complete protease cocktail. The supernatant protein extract was incubated with 5 µg of anti-mCherry antibodies at 4°C for 2 h. For immunoprecipitation, 50 µl magnetic beads were washed three times with PBS buffer and incubated with cellular protein extract samples for 3 h at 4°C with constant rotation. Then, the beads were pelleted down by centrifugation, washed, resuspended in 40 µl of SDS sample loading buffer, denatured at 95°C for 6 min, and analyzed by Western blotting as previously described.

### ELISA assay for estimating insulin levels

INS-1 cells were first pre-incubated with Krebs-Ringer bicarbonate buffer (KRBH; pH 7.4; 114 mM NaCl, 4.7 mM KCl, 1.2 mM KH_2_PO_4_, 1.16 mM MgSO_4_, 0.5 mM MgCl_2_, 2.5 mM CaCl_2_, 0.25% BSA, and 20 mM HEPES) containing 2.8 mM glucose for 1 h. Then, the cells were incubated with KRBH buffer containing 16.7 mM glucose for 30 min. The cell supernatants were collected and analyzed using ELISA kits for Insulin estimation (ImmunoDiagnostics Limited, China) according to the manufacturer’s instructions. For the secretion of insulin in vitro, the pancreas was digested with collagenase P (Roche), then the islets were taken by hand under a dissection microscope. The freshly isolated mouse islets were cultured in RPMI medium containing 10% FBS, 2 mM glutamine for 24 h and then infected by lentivirus for another 48h. Following GSIS, incubation medium was detected by ELISA assay as described above.

### The package and concentration of Lentivirus

293T cells were co-transfected with pCDH-GFP, pmd2g, Pxpax. After 48–72 h of transfection, the supernatant is collected and filtered with a 0.45µm filter (Millipore). Lentivirus followed by centrifugation with Ultracel-100 regenerated cellulose membrane (Millpore) at 5000 rpm for 15 min at 4°C to obtain the concentrate.

### Statistical analysis

The statistical data is reported as mean ± SEM. The statistical differences between groups was estimated using two-tailed Student’s *t-*test or among multiple groups using two-way ANOVA. *P*<0.05 was considered statistically significant.

## Data Availability

Materials and raw data are available from the authors upon request

## Abbreviations

GSIS: glucose-stimulated insulin secretion
PBST: phosphate-buffered saline with Tween-20
PM: plasma membrane
PVDF: polyvinylidene fluoride
SG: secretory granule
SNAP: synaptosome-associated protein
SNARE: Soluble N-ethylmaleimide-Sensitive Factor Attachment Protein
VAMP: vesicle-associated membrane protein

## References

[1] Kadkova A., Radecke J. and Sorensen J.B. (2019) The SNAP-25 Protein Family. Neuroscience 420, 50–71 10.1016/j.neuroscience.2018.09.020 30267828

[2] Baker R.W. and Hughson F.M. (2016) Chaperoning SNARE assembly and disassembly. Nat. Rev. Mol. Cell Biol. 17, 465–479 10.1038/nrm.2016.65 27301672 PMC5471617

[3] Dingjan I., Linders P.T.A., Verboogen D.R.J., Revelo N.H., Ter Beest M. and van den Bogaart G. (2018) Endosomal and Phagosomal SNAREs. Physiol. Rev. 98, 1465–1492 10.1152/physrev.00037.2017 29790818

[4] Furukawa N. and Mima J. (2014) Multiple and distinct strategies of yeast SNAREs to confer the specificity of membrane fusion. Sci. Rep. 4, 4277 10.1038/srep04277 24589832 PMC3940976

[5] Weber T., Zemelman B.V., McNew J.A., Westermann B., Gmachl M., Parlati F. et al. (1998) SNAREpins: minimal machinery for membrane fusion. Cell 92, 759–772 10.1016/S0092-8674(00)81404-X 9529252

[6] Jahn R. and Scheller R.H. (2006) SNAREs–engines for membrane fusion. Nat. Rev. Mol. Cell Biol. 7, 631–643 10.1038/nrm2002 16912714

[7] Rizo J. (2018) Mechanism of neurotransmitter release coming into focus. Protein Sci. 27, 1364–1391 10.1002/pro.3445 29893445 PMC6153415

[8] Chen Y.A. and Scheller R.H. (2001) Snare-mediated__membrane fusion. Nat. Rev. Mol. Cell Biol. 2, 98–106 10.1038/35052017 11252968

[9] Hong W. (2005) SNAREs and traffic. Biochim. Biophys. Acta 1744, 120–144 10.1016/j.bbamcr.2005.03.014 15893389

[10] Sudhof T.C. and Rothman J.E. (2009) Membrane fusion: grappling with SNARE and SM proteins. Science 323, 474–477 10.1126/science.1161748 19164740 PMC3736821

[11] Mayer A. (2002) Membrane fusion in eukaryotic cells. Annu. Rev. Cell Dev. Biol. 18, 289–314 10.1146/annurev.cellbio.18.032202.114809 12142286

[12] Rothman J.E. (1994) Mechanisms of intracellular protein transport. Nature 372, 55–63 10.1038/372055a0 7969419

[13] Stow J.L., Manderson A.P. and Murray R.Z. (2006) SNAREing immunity: the role of SNAREs in the immune system. Nat. Rev. Immunol. 6, 919–929 10.1038/nri1980 17124513

[14] Pfeffer S.R. (2013) A prize for membrane magic. Cell 155, 1203–1206 10.1016/j.cell.2013.11.014 24315088 PMC4712707

[15] Gaisano H.Y. (2017) Recent new insights into the role of SNARE and associated proteins in insulin granule exocytosis. Diabetes Obes. Metab. 19, 115–123 10.1111/dom.13001 28880475

[16] Kasai H., Takahashi N. and Tokumaru H. (2012) Distinct initial SNARE configurations underlying the diversity of exocytosis. Physiol. Rev. 92, 1915–1964 10.1152/physrev.00007.2012 23073634

[17] Hou J.C., Min L. and Pessin J.E. (2009) Insulin Granule Biogenesis, Trafficking and Exocytosis. Insulin IGFs. Vitamins Hormones 473–506 10.1016/S0083-6729(08)00616-X PMC4324607 19251047

[18] Gaisano H.Y. (2014) Here come the newcomer granules, better late than never. Trends Endocrinol. Metab. 25, 381–388 10.1016/j.tem.2014.03.005 24746186

[19] Wheeler M.B., Sheu L., Ghai M., Bouquillon A., Grondin G., Weller U. et al. (1996) Characterization of SNARE protein expression in beta cell lines and pancreatic islets. Endocrinology 137, 1340–1348 10.1210/endo.137.4.8625909 8625909

[20] Zhu D., Zhang Y., Lam P.P., Dolai S., Liu Y., Cai E.P. et al. (2012) Dual role of VAMP8 in regulating insulin exocytosis and islet beta cell growth. Cell Metab. 16, 238–249 10.1016/j.cmet.2012.07.001 22841572

[21] Ohara-Imaizumi M., Nishiwaki C., Kikuta T., Nagai S., Nakamichi Y. and Nagamatsu S. (2004) TIRF imaging of docking and fusion of single insulin granule motion in__primary rat pancreatic β-cells_ different behaviour of granule motion__between normal and Goto-Kakizaki diabetic rat β-cells. Biochem. J. 1, 381, 13-8 10.1042/BJ20040434 PMC1133756 15128287

[22] Rorsman P. and Renstrom E. (2003) Insulin granule dynamics in pancreatic beta cells. Diabetologia 46, 1029–1045 10.1007/s00125-003-1153-1 12879249

[23] Zhu D., Koo E., Kwan E., Kang Y., Park S., Xie H. et al. (2013) Syntaxin-3 regulates newcomer insulin granule exocytosis and compound fusion in pancreatic beta cells. Diabetologia 56, 359–369 10.1007/s00125-012-2757-0 23132338

[24] Spurlin B.A. and Thurmond D.C. (2006) Syntaxin 4 facilitates biphasic glucose-stimulated insulin secretion from pancreatic beta-cells. Mol. Endocrinol. 20, 183–193 10.1210/me.2005-0157 16099818

[25] Knowles M.K., Barg S., Wan L., Midorikawa M., Chen X. and Almers W. (2010) Single secretory granules of live cells recruit syntaxin-1 and synaptosomal associated protein 25 (SNAP-25) in large copy numbers. Proc. Natl. Acad. Sci. U. S. A. 107, 20810–20815 10.1073/pnas.1014840107 21076040 PMC2996426

[26] Hong W. and Lev S. (2014) Tethering the assembly of SNARE complexes. Trends Cell Biol. 24, 35–43 10.1016/j.tcb.2013.09.006 24119662

[27] Lemons P.P., Chen D. and Whiteheart S.W. (2000) Molecular mechanisms of platelet exocytosis: requirements for alpha-granule release. Biochem. Biophys. Res. Commun. 267, 875–880 10.1006/bbrc.1999.2039 10673384

[28] Willett R., Kudlyk T., Pokrovskaya I., Schonherr R., Ungar D., Duden R. et al. (2013) COG complexes form spatial landmarks for distinct SNARE complexes. Nat. Commun. 4, 1553 10.1038/ncomms2535 23462996 PMC3595136

[29] White K.I., Zhao M., Choi U.B., Pfuetzner R.A. and Brunger A.T. (2018) Structural principles of SNARE complex recognition by the AAA+ protein NSF. Elife 7, 10.7554/eLife.38888 PMC6160233 30198481

[30] Rathore S.S., Bend E.G., Yu H., Hammarlund M., Jorgensen E.M. and Shen J. (2010) Syntaxin N-terminal peptide motif is an initiation factor for the assembly of the SNARE-Sec1/Munc18 membrane fusion complex. Proc. Natl. Acad. Sci. U. S. A. 107, 22399–22406 10.1073/pnas.1012997108 21139055 PMC3012463

[31] Ravichandran V., Chawla A. and Roche P.A. (1996) Identification of a novel syntaxin- and synaptobrevin/VAMP-binding protein, SNAP-23, expressed in non-neuronal tissues. J. Biol. Chem. 271, 13300–13303 10.1074/jbc.271.23.13300 8663154

[32] Kunii M., Ohara-Imaizumi M., Takahashi N., Kobayashi M., Kawakami R., Kondoh Y. et al. (2016) Opposing roles for SNAP23 in secretion in exocrine and endocrine pancreatic cells. J. Cell Biol. 215, 121–138 10.1083/jcb.201604030 27697926 PMC5057288

[33] Sadoul K., Berger A., Niemann H., Weller U., Roche P.A., Klip A. et al. (1997) SNAP-23 is not cleaved by botulinum neurotoxin E and can replace SNAP-25 in the process of insulin secretion. J. Biol. Chem. 272, 33023–33027 10.1074/jbc.272.52.33023 9407084

[34] Vaidyanathan V.V., Puri N. and Roche P.A. (2001) The last exon of SNAP-23 regulates granule exocytosis from mast cells. J. Biol. Chem. 276, 25101–25106 10.1074/jbc.M103536200 11350976

[35] Yang L., Peng X., Li Y., Zhang X., Ma Y., Wu C. et al. (2019) Long non-coding RNA HOTAIR promotes exosome secretion by regulating RAB35 and SNAP23 in hepatocellular carcinoma. Mol. Cancer 18, 78 10.1186/s12943-019-0990-6 30943982 PMC6446409

[36] Klein O., Roded A., Zur N., Azouz N.P., Pasternak O., Hirschberg K. et al. (2017) Rab5 is critical for SNAP23 regulated granule-granule fusion during compound exocytosis. Sci. Rep. 7, 15315 10.1038/s41598-017-15047-8 29127297 PMC5681557

[37] Cardenas R.A., Gonzalez R., Sanchez E., Ramos M.A., Cardenas E.I., Rodarte A.I. et al. (2021) SNAP23 is essential for platelet and mast cell development and required in connective tissue mast cells for anaphylaxis. J. Biol. Chem. 296, 100268 10.1016/j.jbc.2021.100268 33837726 PMC7948755

[38] Wang G., Witkin J.W., Hao G., Bankaitis V.A., Scherer P.E. and Baldini G. (1997) Syndet is a novel SNAP-25 related protein expressed in many tissues. J. Cell Sci. 110, 505–513 10.1242/jcs.110.4.505 9067602

[39] Ravichandran V., Chawla A. and Roche P.A. (1996) Identification of a novel syntaxin- and synaptobrevin/VAMP-binding protein, SNAP-23, expressed in non-neuronal tissues. J. Biol. Chem. 271, 13300–13303 10.1074/jbc.271.23.13300 8663154

[40] Wang C.C., Ng C.P., Lu L., Atlashkin V., Zhang W., Seet L.F. et al. (2004) A role of VAMP8/endobrevin in regulated exocytosis of pancreatic acinar cells. Dev. Cell 7, 359–371 10.1016/j.devcel.2004.08.002 15363411

[41] Karim Z.A., Zhang J., Banerjee M., Chicka M.C., Al Hawas R., Hamilton T.R. et al. (2013) IkappaB kinase phosphorylation of SNAP-23 controls platelet secretion. Blood 121, 4567–4574 10.1182/blood-2012-11-470468 23613522 PMC3668489

[42] Guthrie R.A. and Guthrie D.W. (2004) Pathophysiology_of_Diabetes_Mellitus. Crit. Care Nurs. Q. 27, 113–125 10.1097/00002727-200404000-00003 15137354

[43] Shu Y., Liu X., Yang Y., Takahashi M. and Gillis K.D. (2008) Phosphorylation of SNAP-25 at Ser187 mediates enhancement of exocytosis by a phorbol ester in INS-1 cells. J. Neurosci. 28, 21–30 10.1523/JNEUROSCI.2352-07.2008 18171919 PMC6671161

[44] Ran F.A., Hsu P.D., Wright J., Agarwala V., Scott D.A. and Zhang F. (2013) Genome engineering using the CRISPR-Cas9 system. Nat. Protoc. 8, 2281–2308 10.1038/nprot.2013.143 24157548 PMC3969860

